# Effect of CO_2_ Fractional Laser PreTreatment in Photothermal Therapy Using Ethosome Gold Nanoparticles: A Preliminary Study

**DOI:** 10.1111/jocd.70349

**Published:** 2025-07-14

**Authors:** Dong Hye Suh, Sang Jun Lee, Heesu Kim, Kye Yong Song, Ju Young Jo, Su Ji Chae, Hwa Jung Ryu

**Affiliations:** ^1^ ArumdaunNara Dermatologic Clinic Seoul Korea; ^2^ Yonseinew Dermatology and Laser Clinic Incheon Korea; ^3^ Department of Pathology College of Medicine, Chung‐Ang University Seoul Korea; ^4^ Department of Dermatology Korea University Ansan Hospital Ansan Korea

**Keywords:** acne, CO_2_ fractional laser, ethosome gold nanoparticle

## Abstract

**Background:**

The selective photothermolysis of sebaceous follicles with topically applied gold nanoparticles, followed by light exposure, shows good efficacy for acne treatment. Methods such as sonophoresis or CO_2_ fractional laser can help to deliver gold nanoparticles efficiently. Additionally, ethosomes are introduced to enhance the skin penetration of gold nanoparticles.

**Aims:**

This study aimed to assess the effectiveness and safety of the combination of CO_2_ fractional laser and gold photothermal therapy for acne.

**Patinents/Methods:**

Twenty‐four patients with moderate to severe acne were involved. They were randomly divided into two groups: Group 1 received CO_2_ fractional laser treatment followed by ethosome gold nanoparticle ampule application, while Group 2 applied the ampule with sonophoresis. After ampule application, all patients underwent long‐pulsed Nd:YAG laser treatment (1064 nm wavelength, fluence 4.5 J/cm^2^, frequency 10 Hz, pulse duration 0.3 ms, and spot size 10 mm). Clinical evaluation, biopsy, and blood tests for gold detection assessed the effectiveness and safety of the treatments.

**Results:**

Ethosome gold photothermal therapy demonstrated significant clinical and histological improvements in acne vulgaris among Asian patients, showing no serious adverse effects or systemic absorption of gold nanoparticles.

**Conclusions:**

Patients treated with CO_2_ fractional laser pretreatment before ampule application showed reductions across all acne lesion types, including comedones, and experienced improvement in acne scars.

## 1 Introduction

Photothermal therapy (PTT) has emerged as a promising treatment modality for a wide range of dermatological conditions, including acne vulgaris, a prevalent and often distressing skin disorder [1]. Acne affects millions of individuals worldwide. It manifests through inflammatory lesions, comedones, and, in severe cases, cystic nodules, leading to significant physical and psychological impacts. The pathogenesis of acne is multifactorial, involving overproduction of sebum, hyperkeratinization, inflammation, and proliferation of *Cutibacterium acnes* (*C. acnes*), a bacterium that thrives in an anaerobic environment of clogged hair follicles.

Traditional acne therapies including topical retinoids, antibiotics, and oral isotretinoin have provided substantial benefits. However, they have limitations such as antibiotic resistance, side effects, and patient compliance issues. In this context, photothermal therapy using gold nanoparticles, which utilizes light to generate localized heat capable of targeting specific tissues or microorganisms, offers a novel approach to treating acne by directly impacting *C. acnes* and sebaceous glands while minimizing damage to surrounding healthy tissues.

Recent advances in nanotechnology and laser technology have further enhanced the efficacy of photothermal therapy in dermatology. By employing nanoparticles that can absorb light at specific wavelengths, photothermal therapy can be precisely targeted, destroying *C. acnes* bacteria and reducing sebaceous gland activity. This precision can reduce inflammatory responses and promote faster healing with fewer side effects, making PTT a compelling alternative or adjunct to conventional acne treatments.

Ethosomes are advanced lipid vesicles showing significant potential for transdermal drug delivery [2]. Their unique compositions, such as phospholipids, ethanol, and water, allow them to enhance the permeability of the skin barrier, enabling efficient delivery of active pharmaceutical ingredients through the skin. In this study, ethosomes were used to deliver gold nanoparticles. Methods such as sonophoresis and CO_2_ fractional laser can be used later to enhance skin penetration of ethosome gold nanoparticles. This study aimed to assess the effectiveness and safety of gold photothermal therapy after CO_2_ fractional laser pretreatment for acne.

## 2 Materials and Methods

### 2.1 Patients

Twenty‐four patients with moderate to severe acne who visited the Department of Dermatology, Korea University Ansan Hospital and Arumdaun Nara Dermatologic Clinic between June 2022 and February 2023 participated in this study. The study protocol was approved by the Institutional Review Board (IRB) of Korea University Ansan Hospital (IRB no. 2022AS0124). Before undergoing the procedure, all participants were thoroughly informed about the potential benefits and risks associated with the treatment, and written informed consent was obtained from each patient.

### 2.2 Inclusion and Exclusion Criteria

Patients with a history of keloids, active skin infections, or uncontrolled systemic diseases were excluded from this study. Individuals who had received oral antibiotic treatments within the past 3 months, oral retinoid therapy within the past 6 months, or other laser treatments within the past 3 months were also excluded. Pregnant or lactating individuals were also excluded from this study.

### 2.3 Treatment Protocol

Patients applied a local anesthetic cream (a eutectic mixture of 2.5% lidocaine HCl and 2.5% prilocaine; Taiguk Pharm Co. Ltd., Gyeonggi, Republic of Korea) to their faces for 40 min prior to the procedure. Each patient received three consecutive treatments spaced 2–3 weeks apart. They were randomly assigned to two groups: Group 1 and Group 2. Group 1 patients received CO_2_ fractional laser treatment followed by the application of an ethosome gold nanoparticle ampoule (ETHOSOME PTT, N‐FINDERS, Seoul, Republic of Korea), while Group 2 only applied the ethosome gold nanoparticle ampoule using sonophoresis. After ampoule application, all patients underwent a long‐pulsed Nd:YAG laser treatment (V Laser wavelength, 1064 nm; fluence, 5.0 J/cm^2^; frequency, 10 Hz; pulse duration, 0.3 ms; and spot size, 10 mm with cooling off) in a sweeping technique. Ethosome gold nanoparticle ampoules contain gold and platinum nanoparticles, each sized between 50 nm and 100 nm with a spherical shape. During a single treatment, 2 mL of the ampoule was applied to the entire face. Group 1 received CO_2_ fractional laser treatment (U‐pulse, SNJ, Seoul, Korea) with 5 mJ, Density 1.0 mm (distance between dots), and 1.5 × 1.5 cm^2^ sized square scan. Group 2 received sonophoresis (Bellasonic 2X, LTRA Global, Gyeonggi‐do, Korea) 3/10 MHz, Intensity 5 for 10 min for solution penetration.

### 2.4 Clinical Assessment

All evaluations were conducted by two independent dermatologists who were not involved in this study. Clinical photographs were taken at baseline, before each treatment session, and 2 weeks after the final treatment. These photographs were captured using a digital camera (EOS 80D; Canon KK, Tokyo, Japan) with patients positioned consistently under uniform lighting conditions. Changes in the number of papules, pustules, and comedones were recorded both before and after treatment.

Before each subsequent treatment, patients were asked about any adverse effects they had experienced since the previous session. At the final visit, two blinded dermatologists performed overall assessments for each patient using the following scoring scales to evaluate general improvement:

1. No improvement
2. 1%–24% improvement
3. 25%–49% improvement
4. 50%–74% improvement
5. 75%–100% improvement

Also, a patient survey was conducted on their improvement.

### 2.5 Histopathological and Blood Examination

Punch biopsies were obtained from acne lesions before and after treatment in one patient who consented. These specimens were then subjected to hematoxylin and eosin (H&E) staining. To investigate the systemic absorption of gold nanoparticles, blood tests were conducted 2 weeks after the final treatment for four patients who had provided consent. It was sent to the Korea Institute of Science and Technology for ICP‐MS (Inductively Coupled Plasma Mass Spectrometry).

### 2.6 In Vitro Test of Acrylic Plate

**FIGURE 1:**
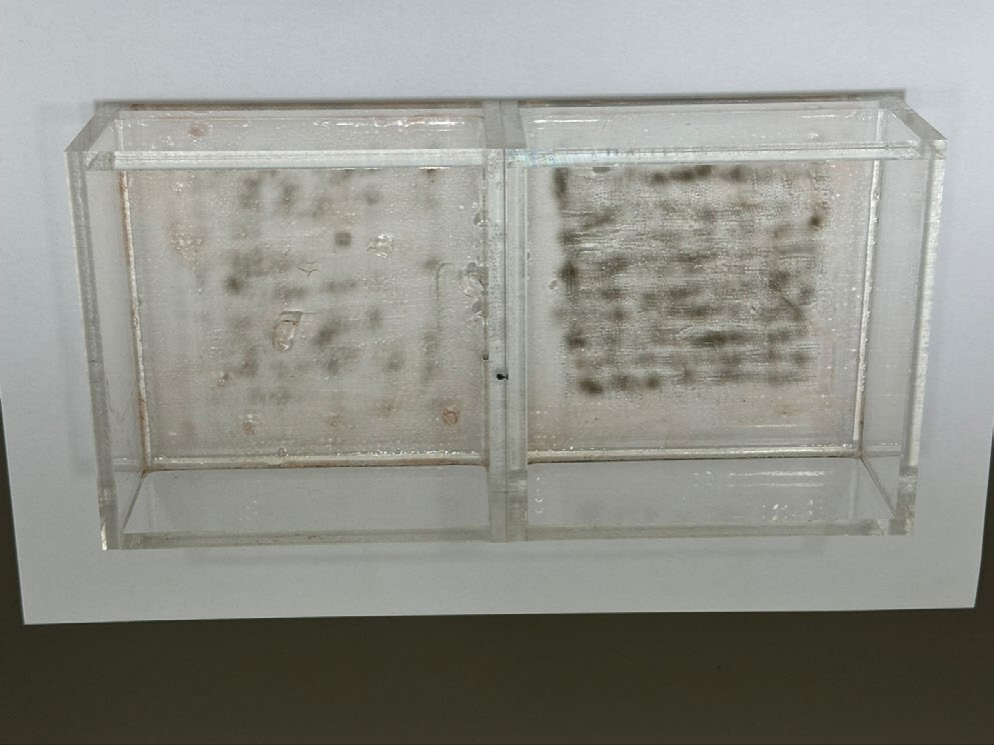
Irradiation with a long‐pulsed Nd:YAG laser after applying gold nanoparticle manually (left) or combining CO_2_ fractional laser and gold nanoparticle application (right).

Ethosome was applied manually onto an acrylic plate. A long‐pulsed Nd:YAG laser was then used for irradiation (Figure 1, left side). CO_2_ fractional laser was used for irradiation before ethosome treatment. A long‐pulsed Nd:YAG laser was then used for irradiation (Figure 1, right side). After CO_2_ fractional laser treatment, ethosome was absorbed much better on the right side than on the left side. Therefore, the reaction was greater after treatment with a long‐pulsed Nd:YAG laser on the right side.

### 2.7 Statistical Analysis

All statistical analyses were conducted using SPSS version 25 software. Changes in the number of lesions, including papules, pustules, and comedones, between baseline and the final treatment were analyzed using the Wilcoxon signed‐rank test. Additionally, a subgroup analysis was performed to compare Group 1 and Group 2 using both paired *t*‐test and the Wilcoxon signed‐rank test. A *p* < 0.05 was considered statistically significant.

## 3 Results

A total of 24 patients completed all treatment sessions and questionnaires. Demographic data for all patients are summarized in Table 1. Of the 24 participants, 11 were males and 13 were females. The mean age of all patients was 21.0 years (range: 14–45 years). All patients had Fitzpatrick skin types III or IV. They were randomly allotted to Group 1 and Group 2, each group having 12 patients.

**TABLE 1:** Demographic data of enrolled patients.

Characteristics	Values
Sex, *n* (%)	
Female	13 (54)
Male	11 (46)
Age, year	
Range	14–45
Mean (SD)	21.0 (5.89)
Fitzpatrick skin type, *n* (%)	
Type III	16 (67)
Type IV	8 (33)

**FIGURE 2:**
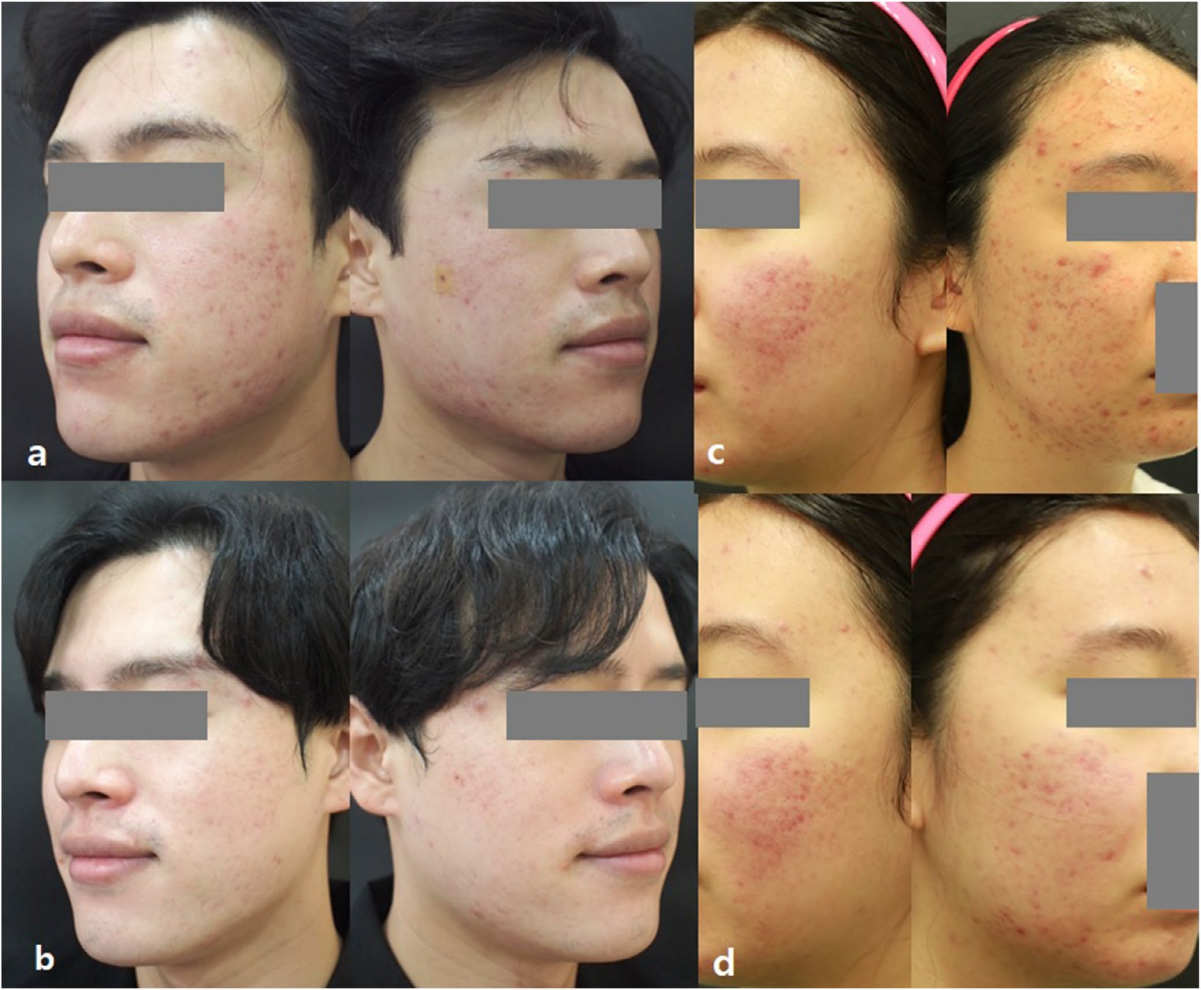
Clinical photos showing improvement in acne lesions after treatment. (a, c) Before treatment. (b, d) After three treatment sessions. The male patient in the left column belonged to Group 1, whereas the female patient in the right column belonged to Group 2.

Objective improvement was observed in all patients, as assessed by dermatologists. Seventeen (71%) patients reported excellent improvement (> 75%) of their conditions (Figure 2).

In statistical analysis of acne lesion improvement, all patients showed a significant reduction in the number of acne lesions (papules, *p* < 0.001; pustules, *p* < 0.001; comedones, *p* = 0.00157) (Table 2). In the subgroup analysis, both Group 1 and Group 2 exhibited decreases in papules and pustules. However, a statistically significant reduction in comedones was observed only in Group 1 (*p* = 0.0043) (Table 3).

**TABLE 2:** Clinical findings at baseline and after the final treatment.

	Baseline, mean (SD)	Final, mean (SD)	% (*p*)
Papule	35.38 (20.89)	14.75 (9.35)	58.31 (< 0.001)^a^
Pustule	12.00 (13.21)	3.63 (6.05)	69.75 (< 0.001)^a^
Comedone	18.12 (17.15)	12.21 (21.40)	32.62 (0.00157)^a^

^a^ Wilcoxon signed‐rank test.

**TABLE 3:** Subgroup analysis of group 1 and group 2 patients.

	Group 1 (CO_2_ fractional)		% (*p*)	Group 2 (Sonopheresis)		% (*p*)
	Baseline, mean (SD)	Final, mean (SD)		Baseline, mean (SD)	Final, mean (SD)	
Papule	32.36 (20.07)	12.86 (8.28)	60.26 (0.0011)^a^	39.60 (22.35)	17.40 (10.54)	56.06 (0.0065)^b^
Pustule	11.86 (11.07)	4.86 (7.63)	59.02 (< 0.001)^b^	12.20 (16.40)	1.90 (1.91)	84.43 (0.0059)^a^
Comedone	13.29 (12.75)	5.79 (5.51)	56.43 (0.0043)^a^	24.90 (20.72)	21.20 (31.19)	14.86 (0.0825)^b^

^a^ Paired *t*‐test.

^b^ Wilcoxon signed‐rank test.

**FIGURE 3:**
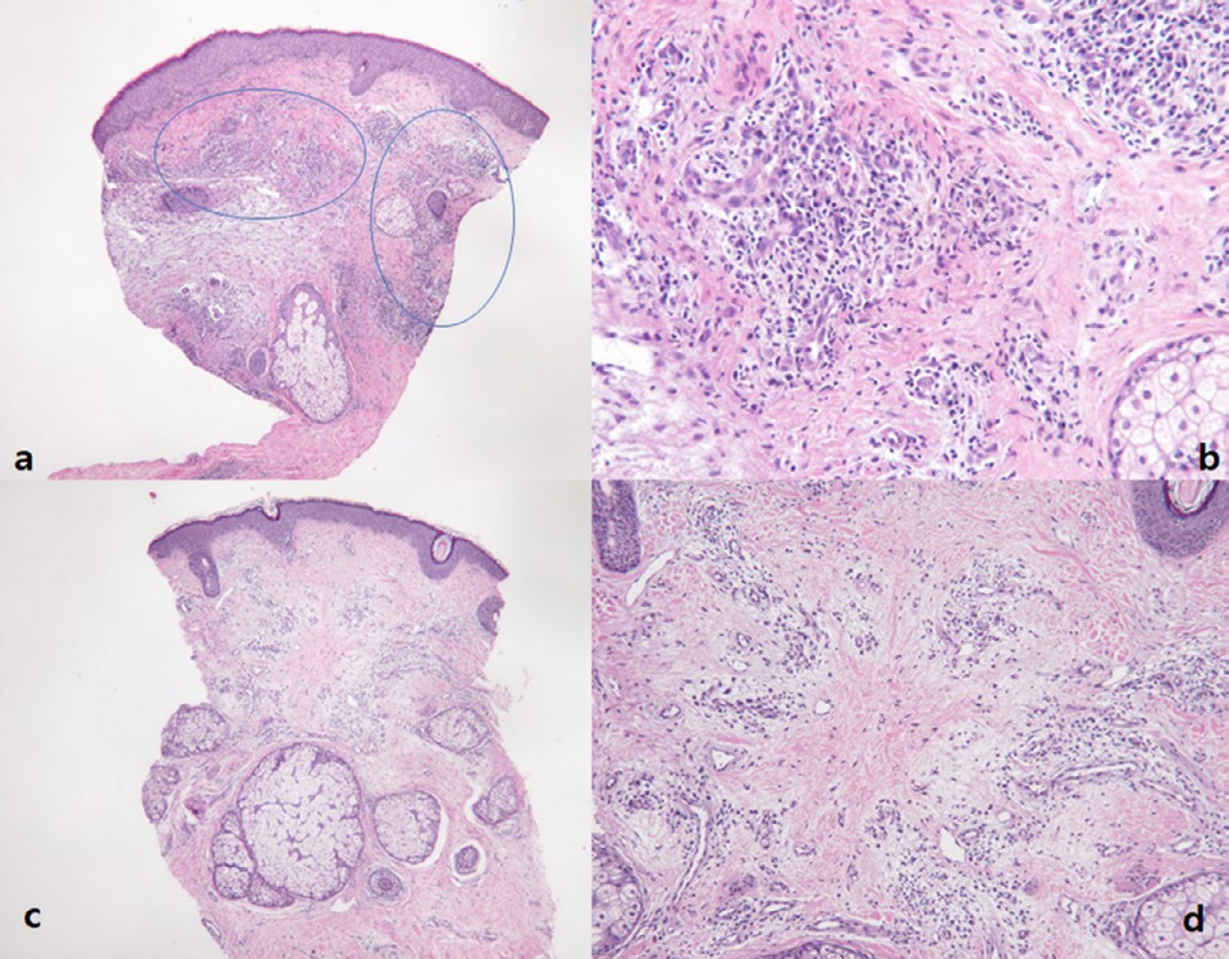
Histology of facial skin before treatment (a, b) and after treatment (c, d) Magnification: ×40 in (a, c); ×100 in (b, d).

One patient had a facial skin biopsy before and after the treatment in Group 1. Histopathological findings using H&E staining revealed decreased inflammatory cell infiltration with fibrotic changes in the dermis following all scheduled treatment sessions (Figure 3).

Four patients underwent blood sampling to analyze systemic absorption of gold nanoparticles. In these patients, gold levels detected in the blood were below the detectable limit (< 3.0 μg/kg).

## 4 Discussion

Acne can be treated using various methods. Recently, alternative treatments have been developed for patients unable to take medications. Among these treatments, selective targeting of sebaceous glands and *Cutibacterium acnes* has gained attention [3]. One such treatment involves a 1726 nm laser that can be highly absorbed by sebaceous glands [4]. Another promising approach is gold photothermal therapy (PTT), which uses gold nanoparticles absorbed into the skin followed by laser irradiation at wavelengths that these particles can absorb well [5].

Gold PTT has been extensively used in cancer therapy [6]. In 2015, Pithankar et al. [1] introduced its application in acne treatment by inducing photothermolysis of sebaceous follicles after absorbing gold microparticles into the skin. Since then, multiple studies have explored gold PTT for acne treatment [5, 7]. However, effective absorption of gold nanoparticles into the skin has been a significant challenge. Research has shown that ultrasound can improve absorption rates compared to simple topical applications or mechanical methods [8]. Using porcine skin, Lee et al. [9] have demonstrated that a combination of thulium laser and sonophoresis can enhance gold nanoparticle delivery, with subsequent long‐pulsed Nd:YAG laser irradiation achieving over 50% destruction of sebaceous follicles by comparing the effects of three methods (sonophoresis alone, thulium laser followed by sonophoresis, and thulium laser followed by manual massage). The most significant sebaceous gland destruction occurred in the group treated with a thulium laser followed by sonophoresis.

Building on this, we used CO_2_ fractional lasers as an alternative to thulium lasers for pretreatment to facilitate nanoparticle absorption. In an in vitro experiment using acrylic plates, pretreatment with CO_2_ fractional lasers followed by long‐pulsed Nd:YAG laser irradiation amplified therapeutic responses. Several techniques, including sonophoresis [10] and iontophoresis [11], have been tested to improve gold nanoparticle absorption. We used CO_2_ fractional laser pretreatment to enhance nanoparticle penetration before applying gold PTT. Sonophoresis is expected to enhance the follicular penetration of gold nanoparticles, whereas CO_2_ fractional laser pretreatment is expected to cause less selective penetration of gold nanoparticles, leading to diffuse dermis heating. This less selectivity may be related to improving diverse acne lesions with CO_2_ fractional laser pretreatment. Further studies are needed with a large number of patients to prove this.

Considering a potential risk of excessive gold absorption, blood gold levels in a few volunteers were measured. Early concerns about systemic absorption of gold nanoparticles emerged during the introduction of gold PTT. Although a review of over 20 studies found no major side effects, it noted the presence of gold in tumor tissues and the liver for up to 174 days [12]. However, these studies were focused on cancer, amyotrophic lateral sclerosis, and relapsing multiple sclerosis, with only one skin‐related research study available. Moreover, differences in the amount of gold nanoparticles used for acne treatment versus cancer therapy remained unclear as reviewed studies did not specify dosages.

Despite initial concerns, Pithankar et al. [1] and others have reported no evidence of systemic absorption. Although intact skin minimally absorbs nanoparticles, CO_2_ fractional laser pretreatment can increase absorption. While this raised concerns about systemic side effects, in vitro tests using acrylic plates confirmed that gold nanoparticle absorption was significantly enhanced following CO_2_ fractional laser pretreatment, amplifying the response to long‐pulsed Nd:YAG lasers. Blood tests in volunteers showed no increase in systemic gold level, alleviating safety concerns.

A recent study elucidated how gold‐based PTT can aid acne treatment [13]. It found that PTT could damage the mitochondrial respiratory chain in sebaceous glands, triggering ferroptosis in sebaceous cells and tissues.

Our clinical trial showed that both sonophoresis and CO_2_ fractional laser methods significantly reduced papules and pustules. However, only those in the CO_2_ fractional laser group achieved a statistically significant reduction in comedones. This suggests that CO_2_ fractional laser pretreatment is more effective across a broader range of acne lesions. Additionally, fractional laser treatment likely contributed to secondary improvements of acne scars. Although pretreatment was intended to reduce procedure time by maximizing absorption, the time spent in wrapping treated areas for occlusion negated these savings. This study is limited by the fact that only one patient's biopsy was used to discuss the histological improvement. Further studies are needed to prove the effects of combining CO_2_ fractional laser and gold‐based PTT on more patients.

## 5 Conclusions

Ethosome gold photothermal therapy demonstrated significant clinical and histological improvements in acne vulgaris among Asian patients, showing no serious adverse effects or systemic absorption of gold nanoparticles. Patients treated with CO_2_ fractional laser pretreatment before ampule application showed reductions across all acne lesion types, including comedones, and experienced improvement in acne scars.

## Author Contributions

D.H.S., S.J.L., and H.J.R. performed the research. D.H.S. and H.J.R. designed the research study. H.K. designed and performed the in vitro study on the acrylic plate. K.Y.S. analyzed the biopsy slides. J.Y.J. analyzed the data. S.J.C. and H.J.R. wrote the paper.

## Ethics Statement

The study protocol received approval from the Institutional Review Board of Korea University Ansan Hospital (IRB no. 2022AS0124). This study complied with the principles outlined in the Declaration of Helsinki.

## Conflicts of Interest

The authors declare no conflicts of interest.

## Data Availability

The authors have nothing to report.
